# Construction and Validation of a Novel Pyroptosis-Related Gene Signature to Predict the Prognosis of Uveal Melanoma

**DOI:** 10.3389/fcell.2021.761350

**Published:** 2021-11-26

**Authors:** Yuan Cao, Jiaheng Xie, Liang Chen, Yiming Hu, Leili Zhai, Jin Yuan, Long Suo, Yaming Shen, Rong Ye, Jiajun Li, Zixuan Gong, Yunfan Dong, Wei Bao, Huan Li, Ming Wang

**Affiliations:** ^1^ The Fourth Clinical Medical College, Nanjing Medical University, Nanjing, China; ^2^ Department of Burn and Plastic Surgery, The First Affiliated Hospital of Nanjing Medical University, Nanjing, China; ^3^ Department of General Surgery, Fuyang Hospital Affiliated to Anhui Medical University, Fuyang, China; ^4^ College of Pharmacy, Jiangsu Ocean University, Lianyungang, China; ^5^ Urology Department, The First Affiliated Hospital of Nanjing Medical University, Nanjing, China; ^6^ Department of Gastroenterology, Nanjing First Hospital, Nanjing Medical University, Nanjing, China; ^7^ Urology Department, The Second Affiliated Hospital of Nanjing Medical University, Nanjing, China

**Keywords:** uveal melanoma, pyroptosis, signature, immune microenvironment, programmed cell death

## Abstract

Uveal melanoma is the most common primary intraocular tumor with a poor prognosis. Currently, treatment for UVM is limited, and the development of drug resistance and tumor recurrence are common. Therefore, it is important to identify new prognostic biomarkers of UVM and explore their role in the tumor microenvironment. Pyroptosis is a way of cell programmed death, and related research is in full throttle. However, the role of pyroptosis in UVM is unclear. In this study, we constructed the prognosis model of pyroptosis-related genes of UVM. This model can accurately guide the prognosis of UVM, and different groups differ in immune infiltration. We further verified our results in cell experiments. To some extent, our study can provide new ideas for the diagnosis and treatment of UVM.

## Introduction

Uveal melanoma is a general term for melanoma that occurs in the choroid, iris, and ciliary body (Chattopadhyay et al., 2016; Kaliki and Shields, 2017). The number of new UVM cases in the United States is 1,500 per year (Smit et al., 2020). Although the total number of cases is small, UVM is the most common primary intraocular malignancy whose most common clinical manifestation is painless vision loss (Luke et al., 2020). When the tumor is large, serous retinal detachment is often associated (Kaliki and Shields, 2017). Once metastasis has occurred, the prognosis for UVM is often poor and treatment options are limited (Carvajal et al., 2018). Treatment options for advanced cutaneous melanoma, such as targeted therapy and immunotherapy, seem to be attractive options for advanced UVM (Jager et al., 2020). Although these treatments are often effective, these treatments are also limited (Jager et al., 2020). It is because that a significant proportion of UVM patients develop drug resistance during treatment, which often leads to tumor recurrence and patient death. Hence, it is time to look for new prognostic indicators of UVM and explore its implications for cancer treatment.

Recently, programmed cell death has become a hot topic in biology (Kovacs and Miao, 2017; Chen et al., 2021a). Pyroptosis is a mode of programmed cell death that plays an important role in homeostasis regulation as well as disease occurrence (Tang et al., 2020; Chen et al., 2021b; Chen et al., 2021c). Pyroptosis is a gasdermin (GSDM) dependent process of membrane perforation accompanied by exudation of inflammatory contents (Xie et al., 2021a). In cancer, pyroptosis has a dual effect. On the one hand, we can reduce tumor load by inducing cancer cells pyroptosis (Xia et al., 2019; Ruan et al., 2020); On the other hand, inflammatory substances released by pyroptosis are involved in the formation of the tumor microenvironment (Jiang et al., 2020). Our current understanding of pyroptosis is far from sufficient, especially in UVM. Pyroptosis is a promising research field.

Here, we combine bioinformatics with cellular experiments to provide a prognostic signature of genes associated with pyroptosis for UVM. Our findings can provide some new ideas for the diagnosis and treatment of UVM.

## Materials and Methods

### Information Extraction of Datasets

We downloaded uveal melanoma RNA sequencing data from TCGA database (https://portal.gdc.cancer.gov/). Data inclusion criteria were: 1) patients diagnosed with uveal melanoma; 2) Patients had detailed mRNA expression and clinical information. Collectively, 80 patients met the inclusion criteria, the gene expression of whom were downloaded for further analysis. (Those with a follow-up of fewer than 30 days were excluded).

### Identification of Genes Associated With Cell Pyroptosis

81 pyroptosis-related genes were extracted from GENECARDS (https://www.genecards.org/). Setting “pyroptosis” as a key word, we searched for pyroptosis-related protein coding genes in the Genecard database. Protein coding genes with correlation score >0.6 were included in our subsequent analysis. A total of 81 genes were eventually included in our analysis.

### Identification of Prognostic Pyroptosis-Related Genes

We initially performed a univariate Cox regression to screen for those potentially prognostic pyroptosis-related genes derived from the TCGA data using R software (version 4.1.0). Packages “survival” were utilized for cox regression analysis. Using R packages “glmnet”, Lasso regression was subsequently performed with those genes that are significantly correlated with patient survival (with *p* < 0.05).

Risk scores were calculated based on the LASSO regression results. The scoring formula is: risk score = $\overset{n}{\underset{i=1}{\sum }}\beta_{i}$ *(expression of pyroptosis associated gene_i)_. Using a median risk score, patients were divided into high-risk and low-risk groups. The defined groups were respectively analyzed during further studies.

### Construction of the Prognostic Model

After the division of patients into high-risk and low-risk groups, survival analysis was performed for both groups to identify the prognostic value of the model. Survival analysis was performed within various subgroups, such as gender, age, and conditions of tumor staging. The accuracy of the established prognosis model was verified by the calibration curve and ROC curve on 1, 3, and 5 years basis.

### Clinical Prediction Value of the Established Prognostic Model

We used univariate and multivariate Cox regressions, respectively, to test whether risk score and clinical characteristics (age, sex, stage) were effective prognostic indicators for uveal melanoma patients. Using R package “forestplot”, the results of cox regression were visualized to see whether the risk score model we previously established was an independent prognostic factor.

### Gene Ontology and Kyoto Encyclopedia of Genes and Genomes Analysis

Differential genes from high-risk and low-risk groups were then subjected to Gene Ontology (GO) and Kyoto Encyclopedia of Genes and Genomes (KEGG) enrichment analysis using R packages “ClusterProfiler” (version 3.0.4), the significantly enriched pathways and ontologies and the associated genes of which were illustrated. Gene Set Enrichment analysis (GSEA) was used as the next enrichment analysis. GSEA required a preexisting set of biologically significant genes (like genes in a pathway), and then the genes in the set (with the same meaning/function) were calculated and summarized into a single enrichment score. This analytical approach added to the interpretability and was used in this study to assess changes in the activity of the pathway/function of the gene set and to select the gene set with *p* < 0.05. Gene Set Variation Analysis (GSVA) was one of the GSEA algorithms. In this study, we collected genes related to immune function and obtained the score of each sample’s immune function Gene Set by GSVA calculation.

### Analysis of Immune Microenvironment

By enquiry into TIMER database (http://timer.cistrome.org/), we downloaded 7 kinds of algorithms of each patient’s immune infiltration situation. Then we analyzed the expression of immune cells in the high and low-risk groups, and isolated the cells with differential expression (*p* < 0.05), before developing a heatmap, At the same time, we studied the expression of immune checkpoint genes between the high and low-risk groups. Similarly, immune checkpoint genes with different expressions (*p* < 0.05) were extracted and the boxplot was made. In DREIMT database (http://www.dreimt.org/), we further analyzed the correlation between the model and immune cells by inputting 80 down-regulated genes and the first 199 up-regulated genes in ascending order of *p*-value into the website. Patients with UVM were scored using the “ESTIMATE” R package to obtain tumor purity score, immune score, stromal score, and total score for each patient.

### Potential Drug Candidate Prediction Using R Software and t*he Construction of a Nomogram*

Using the “pRRophetic” package and the expression matrix of gastric cancer patients, we predicted the minimum drug inhibition concentration (IC_50_) of drugs in uveal melanoma patients of high-risk and low-risk groups, and finally obtained drugs that have statistically different IC_50_ values and may become candidates for the treatment of uveal melanoma. A nomogram of the patient “TCGA-VD-A8KH” was plotted using the “Regplot” package to integrate risk groups with clinical features.

### Cell Culture and Transfection

The human invasive uveal melanoma cell line (MuM2B) was purchased from Fuheng Biology Inc. (Fuheng, Shanghai, China). MUM2B cells were cultured in Roswell Park Memorial Institute 1,640 (RPMI1640, Gibco, Carlsbad, CA, United States), supplemented with 10% Fetal Bovine Serum (Gibco), along with 100 U/mL penicillin and 100 µg/ml streptomycin (Gibco). The cells were cultured in an atmosphere of 5% CO_2_ and at a temperature of 37°C. 24 h prior to transfection, MUM2Bs were seeded onto six-well tissue culture plates at a density of 50,000 cells per well. Cells were starved in 0.5% FBS medium for 6 h before any further treatment.

The small interfering RNA (siRNA) probe for ANO6 and GAPDH positive control oligonucleotides and its control siRNAs were synthesized by Shanghai GenePharma Inc. (Shanghai, China). The sequence of the siRNAs were illustrated in (Supplementary Table S1). Lipo6000™ Transfection reagent (Beyotime, Nanjing, China) was used for the transfection of siRNA according to the manufacturer’s instructions.

### Quantitative Real-Time Polymerase Chain Reaction (qRT-PCR)

Total cellular RNAs were isolated from cells using Trizol Reagent (Invitrogen, Carlsbad, CA, United States) according to the manufacturer’s instructions. The reverse transcription was conducted using the reverse transcription kit provided by Takara (Otsu, Shiga, Japan). Real-time polymerase chain reaction (RT-PCR) was performed using a QuantiTect SYBR Green PCR Kit (Takara), and on a Applied Biosystems QuantStudio 1 (Thermo, Waltham, MA, United States). Relative quantification was determined using the −2ΔΔCt method. The relative expression of messenger RNA (mRNA) for each gene was normalized to the level of glyceraldehyde-3-phosphate dehydrogenase (GAPDH) mRNA. The primers were synthesized by GenePharma Inc. (Shanghai, China), the sequence of which were listed in (Supplementary Table S2).

### Cell Proliferation and Cell Cycle Analysis

5-ethynyl-2 deoxyuridine (EdU) assay was performed according to the manufacturer’s instructions (Beyotime, Shanghai, China), MUM2B cells were incubated with EdU for 2 h. The number of proliferating cells were analyzed under an Olympus confocal microscope (Olympus, Tokyo, Japan).

### Cell Migration Assay

A scratch wound-healing migration assay was performed in transfected and non-transfected uveal melanoma cell lines to validate the relationship between the prognostic genes and the tumor cell migration ability. When the MUM2Bs reached 90–100% confluence in the 6-well culture plate, cells were subjected to serum-free RPMI1640 medium for 24 h. After serum starvation, one line within the MUM2Bs were scraped using a sterile plastic pipette tip in each cultured well. The cells were washed twice in warm serum-free medium to remove cellular debris After 0, 6, and 12 h the scratch wounds were subjected to microscope photography. Images were acquired using a microscope (Olympus, Tokyo, Japan), and cell migration was determined by the percentage of the wound closure area in five independent experiments using the Image J software.

### Data Processing and Statistical Analysis

The quantification of the experiment data was conducted using Image J software (version 1.0.3). All experimental data were analyzed using IBM SPSS software (version 16.0.0) multiple comparisons were conducted using one-way ANOVA and Student- Newman-Keuls (SNK) multiple comparison method, and *p* < 0.05 was considered statistically significant.

## Results

### 5 Pyroptosis-Related Genes Were Identified to Calculate the Risk Score

**FIGURE 1:**
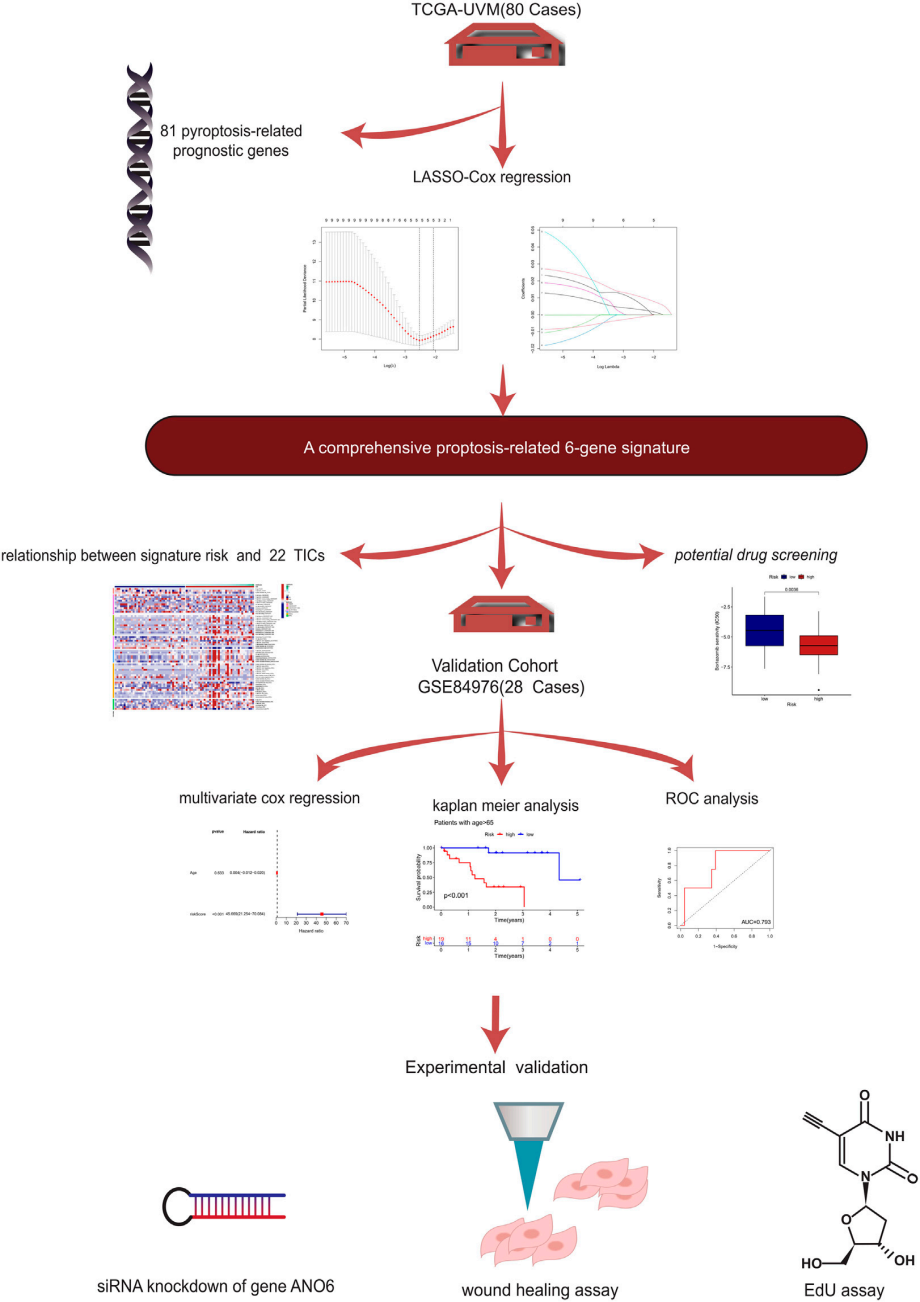
The flow chart.

**FIGURE 2:**
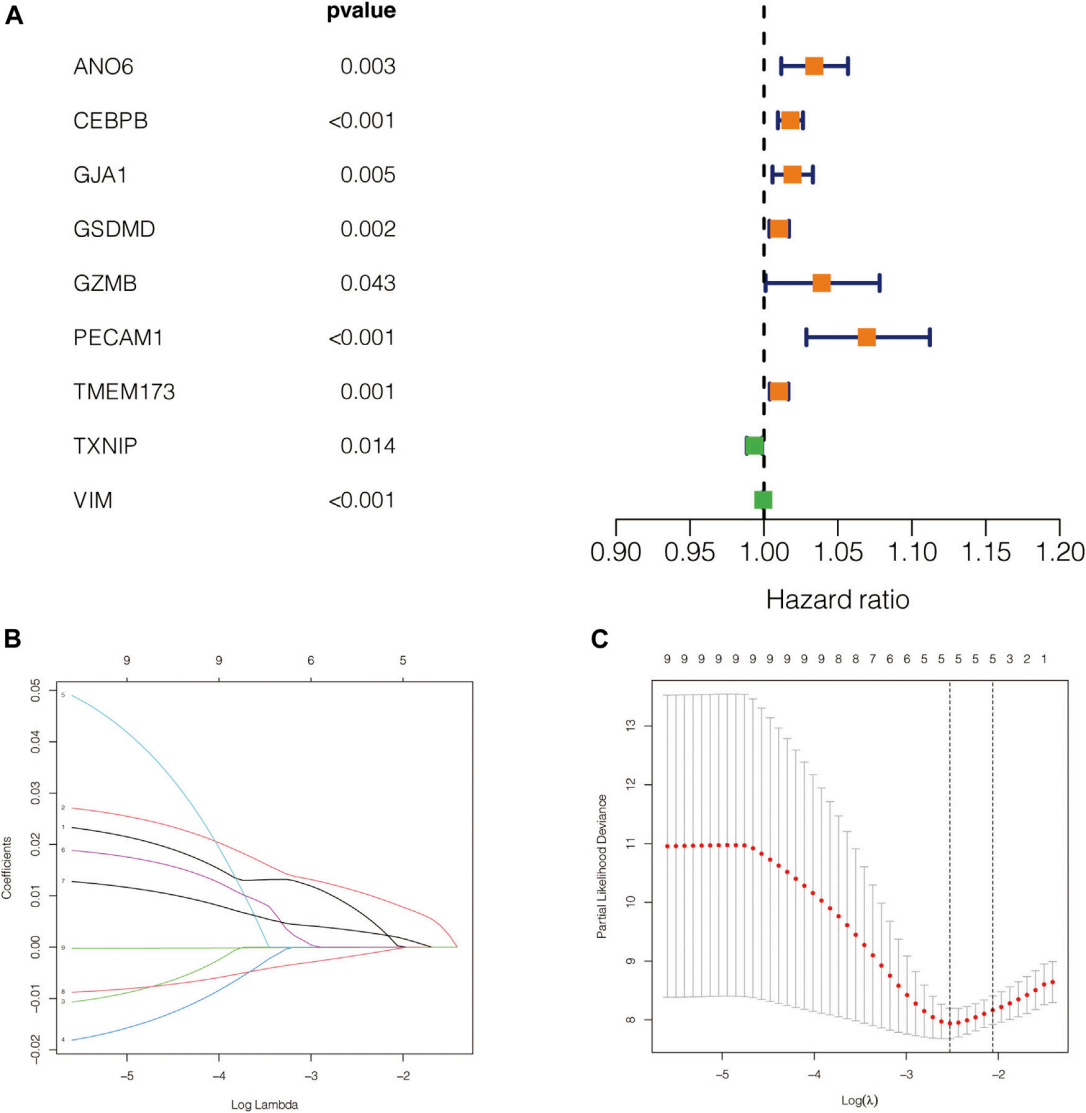
Construction of the prognostic model. **(A)** The forestplot which contained the Hazard Ratio (HR) and its 95% confidence interval. **(B,C)** The potential prognostic genes were subsequently subjected to LASSO-Cox regression in the training cohort to generate a Prognostic signature.

The flow diagram of our present study is illustrated in Figure 1. Based on the previously described inclusion criteria, 80 UVM patients were treated as the training cohort. Using univariate Cox regression, we screened for the genes which are potentially related to the patient’s prognosis (with *p*-value <0.05), both in the training cohort and in the validation cohort as well. An intersection was performed subsequently to achieve a higher confidence level, which included nine potentially prognostic genes. The forestplot which contained the Hazard Ratio (HR) and its 95% confidence interval can be seen in Figure 2A. The potential prognostic genes were subsequently subjected to LASSO-Cox regression in the training cohort to generate a Prognostic signature (Figures 2B,C). 5 pyroptosis-related genes with prognostic value were eventually obtained. The names and the coefficients of the prognostic genes were listed in Table1. The risk score was calculated according to the formula which was previously described.

**TABLE 1:** The names and the coefficients of the prognostic genes.

Gene	Coef
ANO6	0.00750335379446634
CEBPB	0.0110094507963823
TMEM173	0.00320430405522329
TXNIP	−0.00165919015758192
VIM	−9.89378996691334e-05

### Based on the Median Risk Score, 80 Melanoma Patients in TCGA Were Divided Into High-Risk and Low-Risk Groups

The group model based on risk score effectively predicted the prognosis of melanoma patients.

**FIGURE 3:**
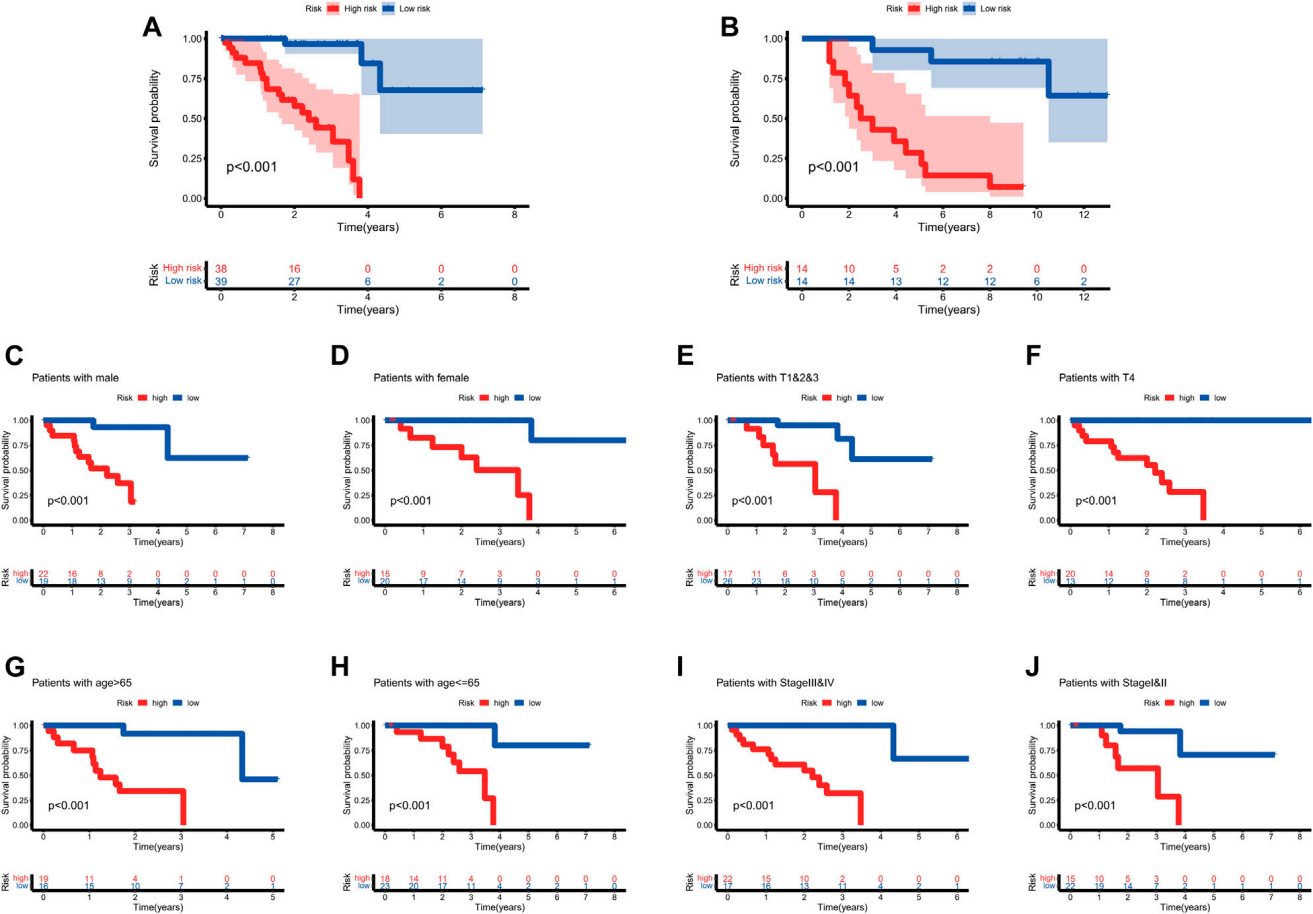
The survival analysis based on the prognostic model. **(A,B)** To further test the confidence of the risk model, a survival analysis was performed between high-risk group and low risk groups between the training cohort (Figure 3A) and the validation cohort (Figure 3B). **(C–J)** The survival rate of patients in the high-risk group suffered a more drastic decrease, regardless of the age, gender, and tumor stages. The differences were statistically significant (*p* < 0.001).

**FIGURE 4:**
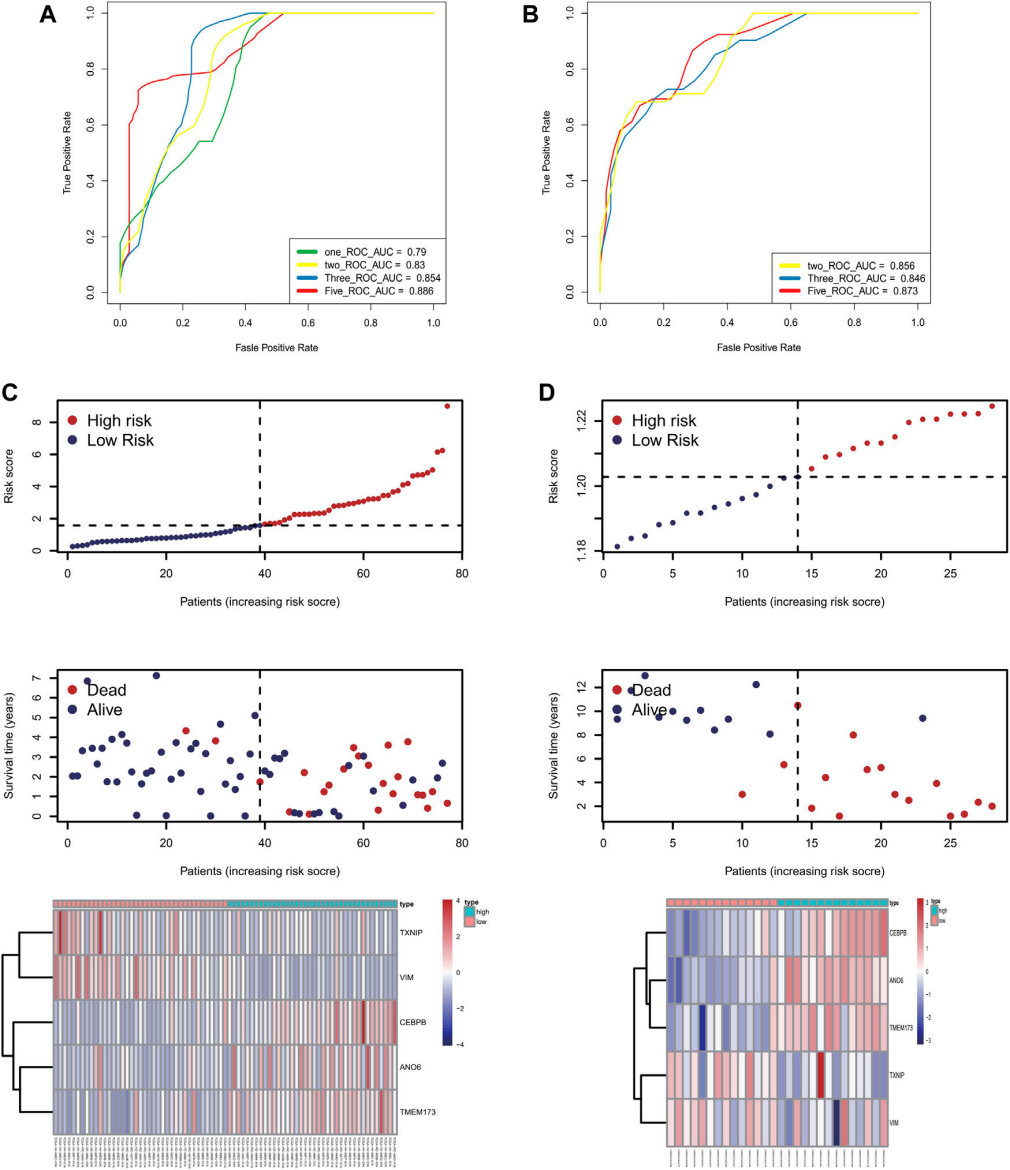
The accuracy of this prognostic model. **(A,B)** The ROC curve of patient’s survival of different years in the training cohort and the validation cohort showed that the model had a potent predicting ability, with the 1-year, 2-years, 3-years and 5-years AUC being 0.79, 0.83, 0.854, 0.886 respectively in the training cohort (Panel 4A), and the 2-years, 3-years and 5-years AUC being 0.856, 0.846, 0.873 respectively in the validation cohort (Panel 4B). (**C,D**) The distribution of the risk scores, outcome status, and gene profiles of the gene signature in the training and validation cohort were shown. The risk group successfully predicted the outcomes of the patients in both the training cohort and the validation cohort, with significantly more events found in the high-risk group. Moreover, the expression of genes ANO6, CEBPB and TMEM173 were upregulated, while genes VIM, TXNIP were seen downregulated in the high-risk group both in the training cohort and in the validation cohort, which was in accordance with our previous risk model.

To further test the confidence of the risk model, a survival analysis was performed between high-risk group and low risk groups between the training cohort (Figure 3A) and the validation cohort (Figure 3B). Meanwhile, a further, more specifically classified survival analysis was performed in different subgroups in the training cohort. The survival rate of patients in the high-risk group suffered a more drastic decrease, regardless of the age, gender, and tumor stages. The differences were statistically significant (*p* < 0.001) (Figures Figure3C–J). The ROC curve of patient’s survival of different years in the training cohort and the validation cohort showed that the model had a potent predicting ability, with the 1-year, 2-years, 3-years and 5-years AUC being 0.79, 0.83, 0.854, 0.886 respectively in the training cohort, and the 2-years, 3-years, and 5-years AUC being 0.856, 0.846, 0.873 respectively in the validation cohort. (Figures 4A,B). The findings revealed that the model was effective in predicting patient’s survival. The distribution of the risk scores, outcome status, and gene profiles of the gene signature in the training and validation cohort were shown in Figures 4C,D. The risk group successfully predicted the outcomes of the patients in both the training cohort and the validation cohort, with significantly more events found in the high-risk group. Moreover, the expression of genes ANO6, CEBPB and TMEM173 were upregulated, while genes VIM, TXNIP were seen downregulated in the high-risk group both in the training cohort and in the validation cohort, which was in accordance with our previous risk model (Figures 4C,D).

### Univariate and Multivariate Cox Regression Revealed That the Risk Score Was an Independent Prognostic Factor in UVM Patients

**FIGURE 5:**
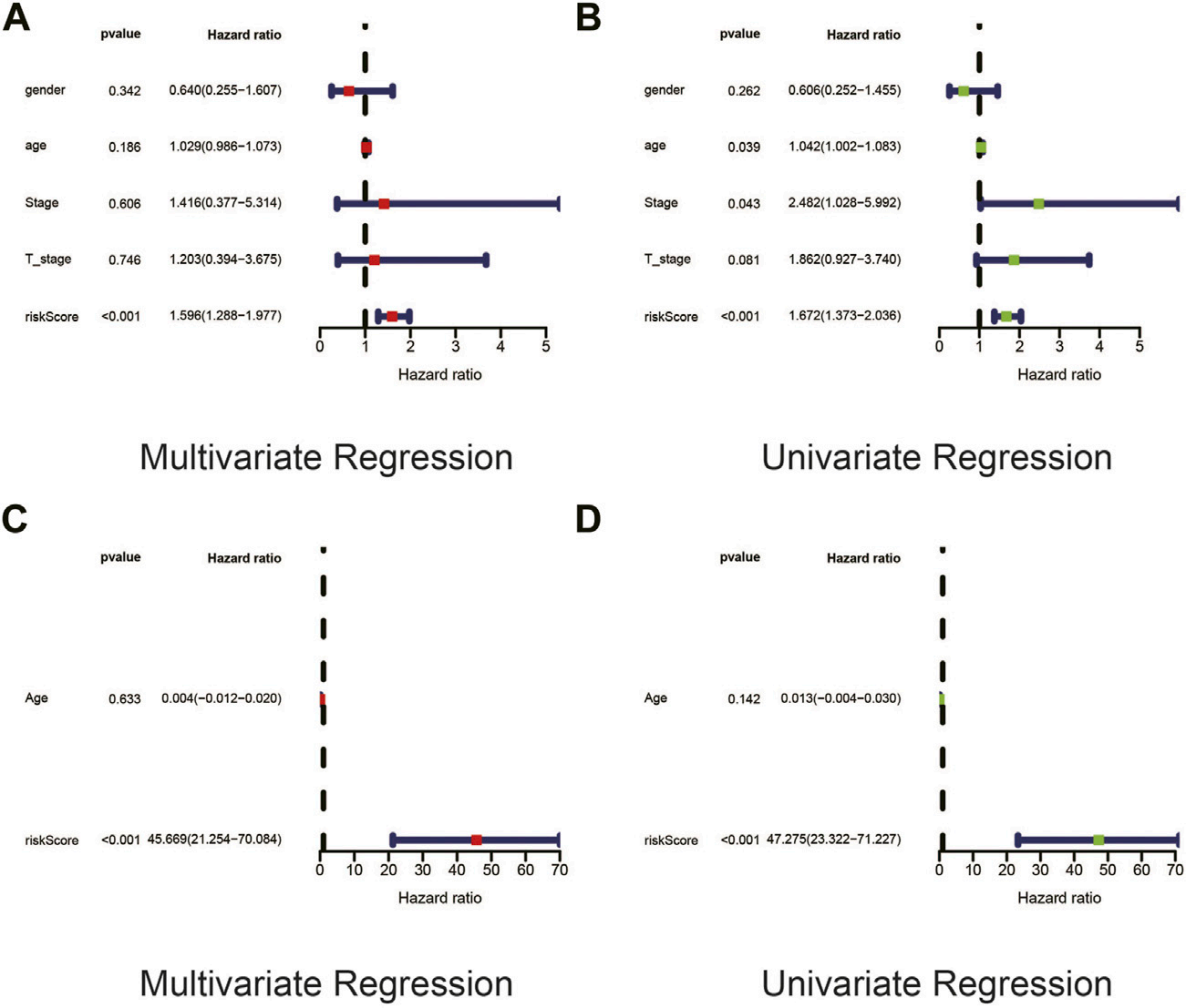
Univariate and Multivariate Cox regression revealed that the risk score was an independent prognostic factor in UVM patients. **(A,B)** In order to explore whether risk score was an independent influencing factor for melanoma patients, univariate and multivariate Cox regression were performed for risk score, age, gender, TNM stage and other factors in both the training cohort and the validation cohort. Cox regression showed that risk score was a independent prognostic factor using univariate cox regression using both univariate (Panel 5A) and multivariate (Panel 5B) regression, despite the fact that tumor stage could also be considered as an independent prognostic factor by means of univariate cox regression. (**C,D**) Likewise, risk score was also an independent prognostic factor with statistical significance in validation cohort.

In order to explore whether risk score was an independent influencing factor for melanoma patients, univariate and multivariate Cox regression were performed for risk score, age, gender, TNM stage and other factors in both the training cohort and the validation cohort. Cox regression showed that risk score was a independent prognostic factor using univariate cox regression using both univariate and multivariate regression (Figures 5A,B), despite the fact that tumor stage could also be considered as an independent prognostic factor by means of univariate cox regression. Likewise, risk score was also an independent prognostic factor with statistical significance in validation cohort (Figures 5C,D).

### Gene Ontology and Kyoto Encyclopedia of Genes and Genomes Enrichment Analysis Revealed That the Differential Genes Between High Risk and Low Risk Groups Were Strongly Correlated With Immune Response

**FIGURE 6:**
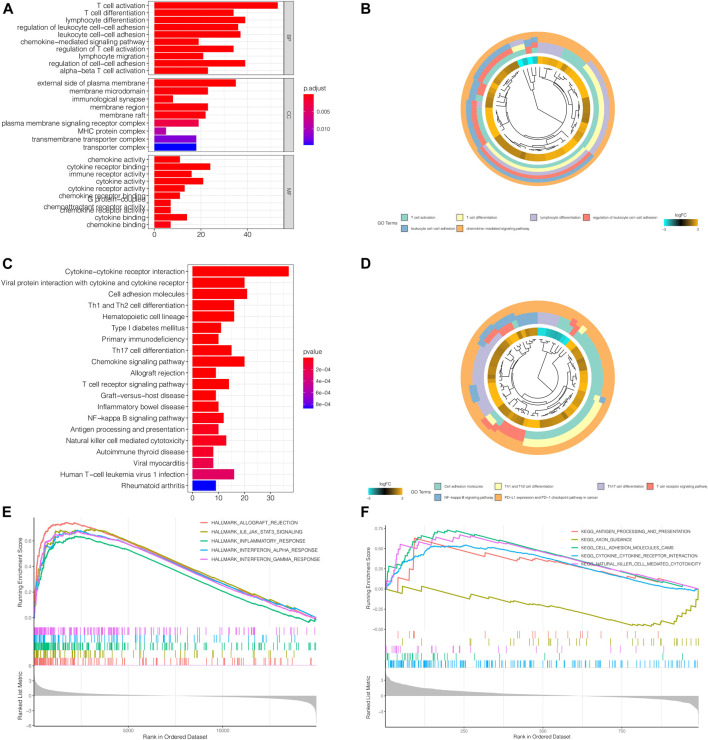
GO and KEGG Enrichment Analysis revealed that the differential genes between high risk and low risk groups were strongly correlated with immune response. **(A,B)** GO Enrichment analysis revealed that, biological processes like T cell activation and differentiation, lymphocyte differentiation and leukocyte cell-cell adhesion; molecular functions such as chemokine activity, cytokine activity, immune receptor activity, as well as cytokine receptor activity were enriched in differential genes between the high-risk group and the low-risk group. The enriched genes were mainly concentrated in the external side of the plasma membrane, which was in line with our findings that the differential genes were correlated immune response. **(C,D)** KEGG analysis revealed a more specific picture of which pathways were enriched between the two groups. Not only was Th1 and Th2 cell differentiation related to the risk model, but PD-1 and PD-L1 activity was also concerned. However, further studied are still needed to validate the relationship between the relationship with our model and immune response. **(E)** GSEA showed that immune-related functional gene sets were mainly enriched in the high-risk group, including allograft rejection, IL6-JAK-STAT3 signaling pathway, inflammatory response, interferon-alpha response, interferon-gamma response. **(F)** 2,483 immune-related genes were downloaded from IMMPORT database. Through GSEA analysis, we found that these immune-related genes are mainly enriched in antigen processing and presentation, cell adhesion molecules, cytokine receptor interactions, and ccytotoxicity mediated by natural killer cells in the high-risk group. Axon guidance was the main enrichment pathway in the low-risk group.

GO Enrichment analysis revealed that, biological processes like T cell activation and differentiation, lymphocyte differentiation and leukocyte cell-cell adhesion; molecular functions such as chemokine activity, cytokine activity, immune receptor activity, as well as cytokine receptor activity were enriched in differential genes between the high-risk group and the low-risk group. The enriched genes were mainly concentrated in the external side of the plasma membrane, which was in line with our findings that the differential genes were correlated immune response. (Figures 6A,B). KEGG analysis revealed a more specific picture of which pathways were enriched between the two groups. Not only was Th1 and Th2 cell differentiation related to the risk model, but PD-1 and PD-L1 activity was also concerned (Figures 6C,D). GSEA showed that immune-related functional gene sets were mainly enriched in the high-risk group, including allograft rejection, IL6-JAK-STAT3 signaling pathway, inflammatory response, interferon-alpha response, interferon-gamma response (Figure 6E). Next, 2,483 immune-related genes were downloaded from IMMPORT database. Through GSEA analysis, we found that these immune-related genes are mainly enriched in antigen processing and presentation, cell adhesion molecules, cytokine receptor interactions, and cytotoxicity mediated by natural killer cells in the high-risk group. Axon guidance was the main enrichment pathway in the low-risk group (Figure 6F).

### Analysis of Immune Microenvironment

**FIGURE 7:**
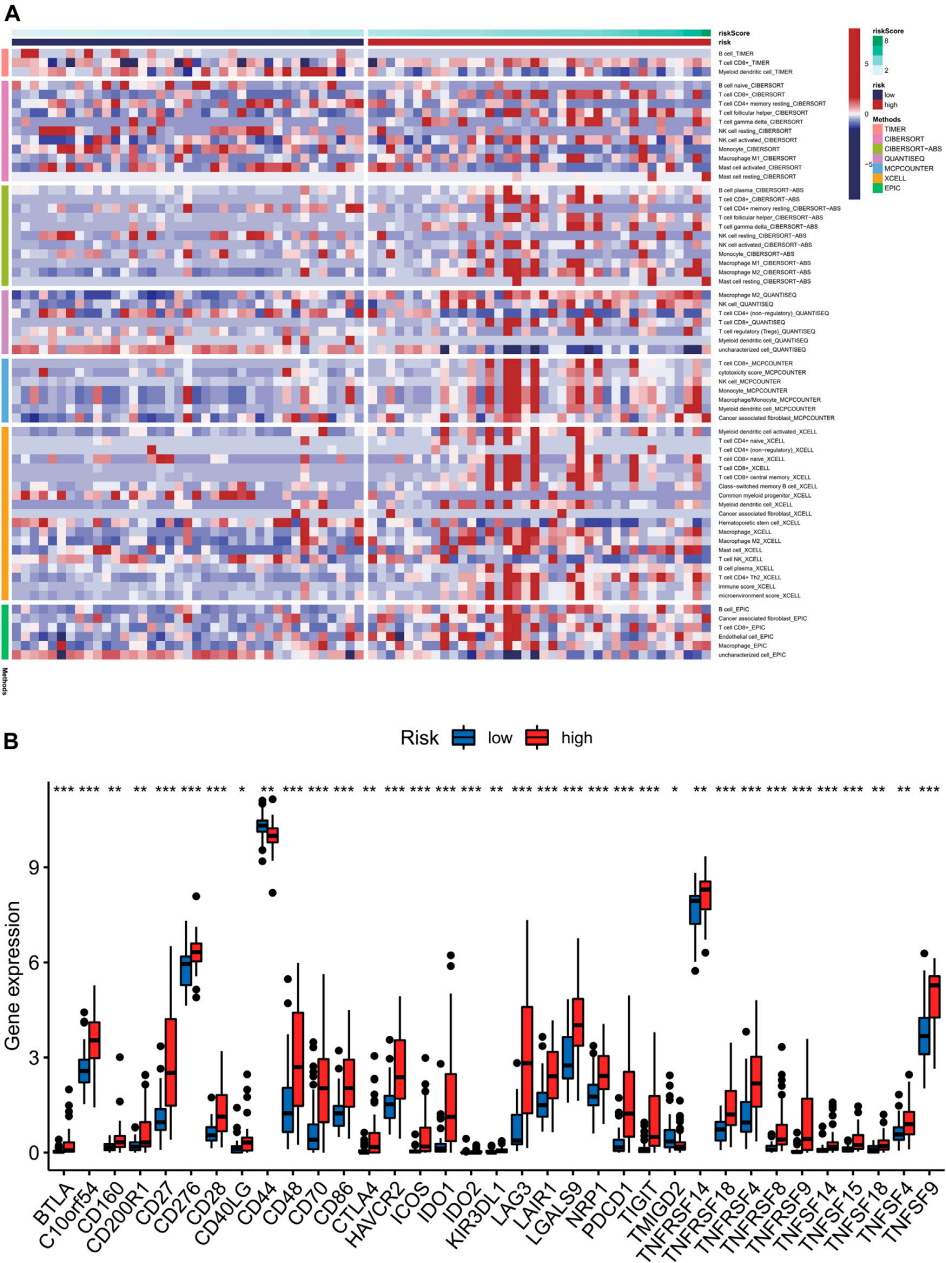
Analysis of immune cell infiltration levels correlation analysis of immune checkpoint and in different risk groups. **(A)** The samples in the training cohort were displayed according to ascending risk scores, while the levels of immune infiltration in high-risk and low-risk groups were calculated by algorithm such as TIMER, CIBERSORT, Quantiseq and other methods, which were marked in different colors. The heatmap showed the level of each immune cell infiltration in the tumor microenvironment of the two groups of patients under different algorithms. **(B)** We could see that the high-risk group tended to have higher levels of immune cells in the tumor microenvironment. Likewise, The immune checkpoint genes were expressed differently in the two groups and gene tended to be higher in the high-risk group (***p < 0.001).

**FIGURE 8:**
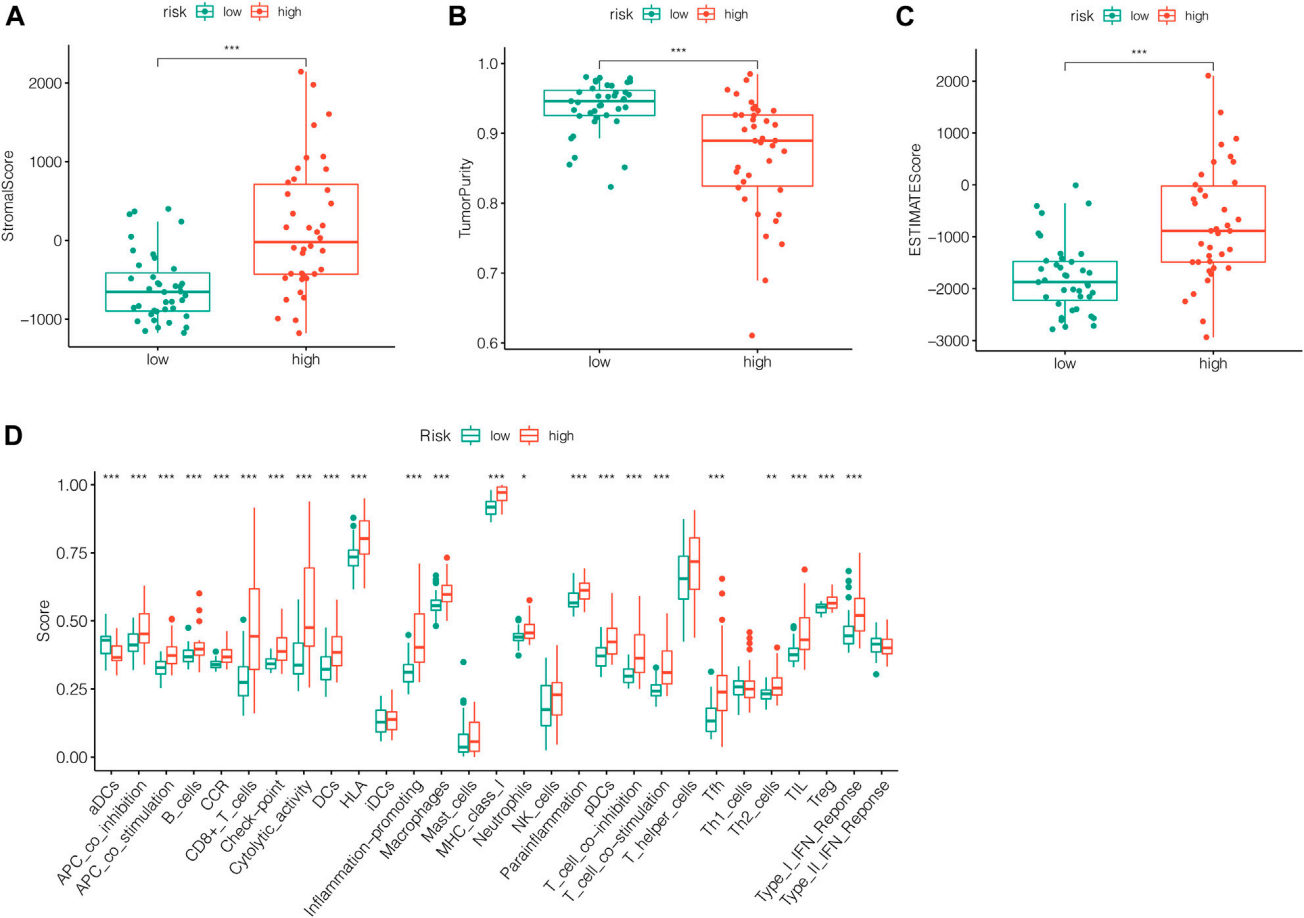
Analysis of immune score between two groups. **(A)** The stromal score was higher in the high-risk group than in the low-risk group (*p* < 0.001). **(B)** Patients in the high-risk group had lower tumor purity scores than those in the low-risk group (*p* < 0.001). **(C)** The ESTIMATE score was higher in the high-risk group than in the low-risk group (*p* < 0.001). **(D)** The total immune score of patients in the high-risk group showed a higher trend.

To further analyze the relationship between tumor immune response and the risk model we previously constructed, immune infiltration analysis using various methods was conducted. The levels of immune infiltration differed between different risk groups. In Figure 7A, the samples in the training cohort were displayed according to ascending risk scores, while the levels of immune infiltration in high-risk and low-risk groups were calculated by algorithm such as TIMER, CIBERSORT, Quantiseq and other methods, which were marked in different colors. The heatmap showed the level of each immune cell infiltration in the tumor microenvironment of the two groups of patients under different algorithms. We could see that the high-risk group tended to have higher levels of immune cells in the tumor microenvironment. Likewise, The immune checkpoint genes were expressed differently in the two groups and gene tended to be higher in the high-risk group (***p < 0.001) (Figure 7B). In Figures 8A–D, we can see the differences in stromal score, tumor purity, ESTIMATE score and total score between the two groups (***p < 0.001). The stromal score and ESTIMATE score were higher in the high-risk group than in the low-risk group (*p* < 0.001). Patients in the high-risk group had lower tumor purity scores than those in the low-risk group (*p* < 0.001). In terms of total score, the score of patients in the high-risk group showed a higher trend.

### Analysis of Sensitivity Difference of Antitumor Drugs in Different Groups and the Construction of a Nomogram

**FIGURE 9:**
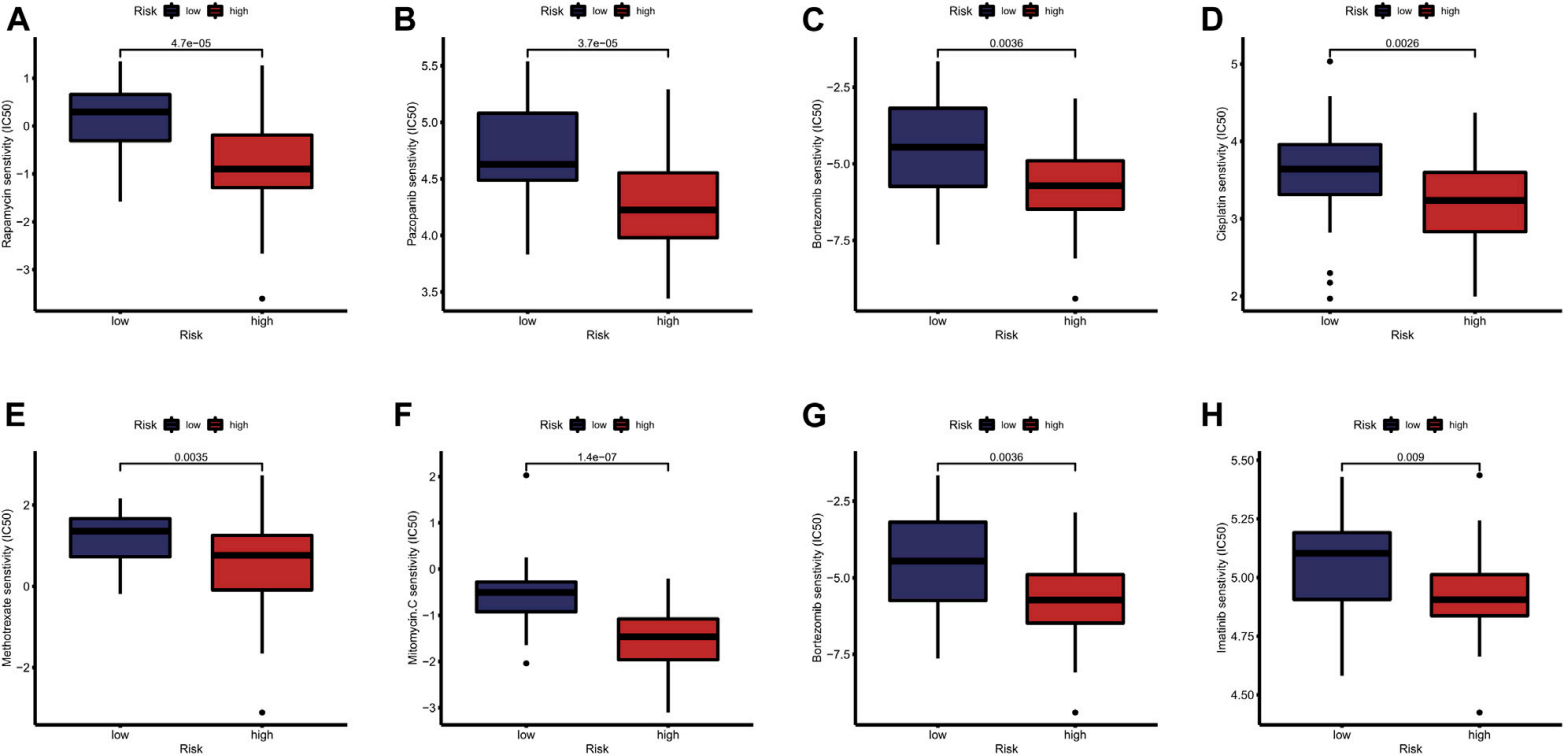
Analysis of sensitivity difference of antitumor drugs in different groups. **(A–H)** The study of the sensitivity of different groups of patients to anti-tumor drugs can provide help for the formulation of future treatment regimens. Boxplot showed the differential IC_50_ of our previously established high-risk and low risk groups in the TCGA cohort. The high-risk group was more sensitive to antitumor drugs like Rapamycin, Pazopanib, Bortezomib, Cisplatin, Methotrexate, Mitomycin. C, Bortezomib and Imatinib. Those antitumor drugs were potentially more capable of inhibiting high-risk uveal melanoma with relatively minor dosage. However, further investigations are required to understand their mechanisms in Uveal Melanoma inhibition.

**FIGURE 10:**
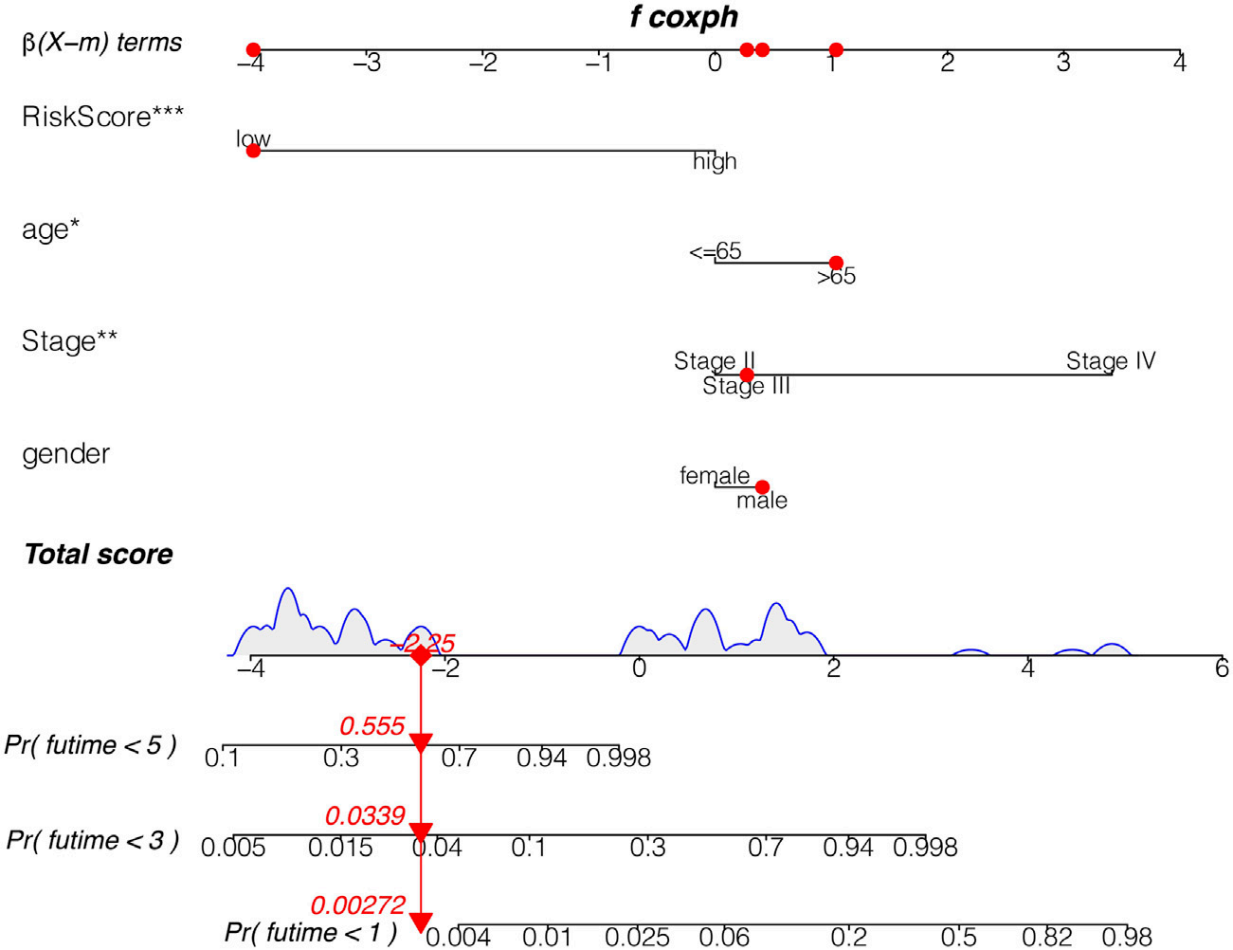
The nomogram. The 1, 3, and 5 mortality rates of patient “TCGA-VD-A8KH” were 0.00272, 0.0339 and 0.555, respectively.

The study of the sensitivity of different groups of patients to anti-tumor drugs can provide help for the formulation of future treatment regimens. Boxplot showed the differential IC_50_ of our previously established high-risk and low risk groups in the TCGA cohort. The high-risk group was more sensitive to antitumor drugs like Rapamycin, Pazopanib, Bortezomib, Cisplatin, Methotrexate, Mitomycin. C, Bortezomib and Imatinib. Those antitumor drugs were potentially more capable of inhibiting high-risk uveal melanoma with relatively minor dosage. However, further investigations are required to understand their mechanisms in Uveal Melanoma inhibition. (Figures 9A–H). To further evaluate the survival of UVM patients, we drew a Nomogram combining the risk value and clinical characteristics of the model. As shown in Figure 10, we found that the 1-, 3-, and 5-years mortality rates of patient “TCGA-VD-A8KH” were 0.00272, 0.0339 and 0.555, respectively.

### ANO6 Knockdown Slows Down Uveal Melanoma Cell Proliferation *in vitro*

**FIGURE 11:**
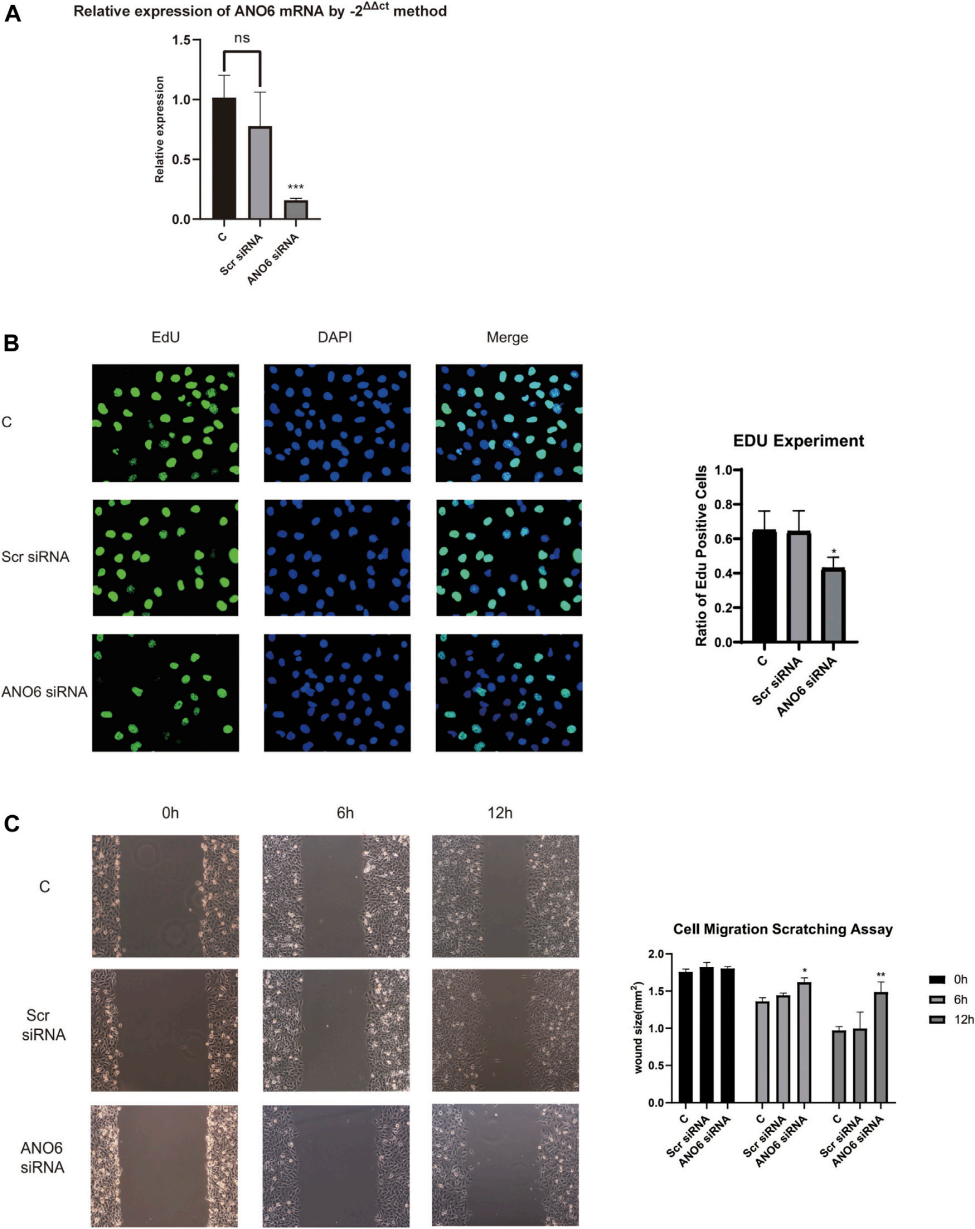
Cell experiments of gene ANO6 to verify our results. **(A)** Firstly, RT-qPCR analysis was performed to validate the knockdown of gene ANO6 mRNA. The level of ANO6 mRNA expression was significantly downregulated after ANO6 siRNA transfection in MUM2B cell lines, which is valid for further investigation (*p* < 0.001). **(B)** 5-ethynyl-2 deoxyuridine (EdU) assay was performed to test whether the knockdown of gene ANO6 could influence uveal melanoma cell proliferation *in vitro*. After knockdown of ANO6 gene, the in MUM2B cell lines showed a significant decrease in nuclear DNA synthesis, indicating the ANO6 gene may progress the proliferation of uveal melanoma cell lines, which sheds light on further study. The results were statistically significant (*p* < 0.05). **(C)** Scratch assays showed that after the knockdown of ANO6 mRNA, MUM2B cells migrated slower than scrambled siRNA or mock-treated control cells, which means that ANO6 knockdown may attenuate the migration of uveal melanoma cells. The results were of statistical significance (*p* < 0.05).

We further performed the experimental analysis of genes that are in the prognostic signature to validate their functions in uveal melanoma cell growth and migration. Because gene ANO6 had a relatively higher level of hazard ratio, and tended to be robust in our previously constructed models, the oncogenic role of ANO6 was tested in further experiments. Firstly, RT-qPCR analysis was performed to validate the knockdown of gene ANO6 mRNA. Figure 11A showed that the level of ANO6 mRNA expression was significantly downregulated after ANO6 siRNA transfection in MUM2B cell lines, which is valid for further investigation (*p* < 0.001).

5-ethynyl-2 deoxyuridine (EdU) assay was performed to test whether the knockdown of gene ANO6 could influence uveal melanoma cell proliferation *in vitro*. After knockdown of ANO6 gene, the in MUM2B cell lines showed a significant decrease in nuclear DNA synthesis, indicating the ANO6 gene may progress the proliferation of uveal melanoma cell lines (Figure 11B), which sheds light on further study. The results were statistically significant (*p* < 0.05).

### ANO6 Knockdown Attenuates Uveal Melanoma Cell Migration *in vitro*

Scratch assays in Figure 11C showed that after the knockdown of ANO6 mRNA, MUM2B cells migrated slower than scrambled siRNA or mock-treated control cells, which means that ANO6 knockdown may attenuate the migration of uveal melanoma cells. The results were of statistical significance (*p* < 0.05).

### The Prognostic Value of This Pyroptosis-Related Gene Signature in Other Cancers

**FIGURE 12:**
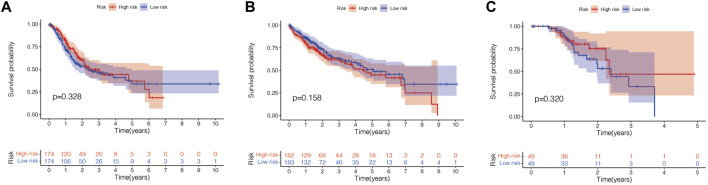
The prognostic value of this pyroptosis-related gene signature in other cancers. (**A**) This signature has no prognostic significance in gastric cancer (*p* = 0.328). (**B**) This signature has no prognostic significance in liver cancer (*p* = 0.158). (**C**) This signature has no prognostic significance in skin melanoma (*p* = 0.320).

Finally, we explored whether this signature also has prognostic significance in other tumors (gastric cancer, liver cancer, skin melanoma). The same method was used to calculate the risk scores of patients with gastric cancer, liver cancer and cutaneous melanoma in the TCGA database, and the patients were divided into the high-risk group and the low-risk group according to the median score value. Then we performed survival analysis between the two groups. As shown in Figures 12A–C, this signature has no prognostic significance in gastric cancer, liver cancer and skin melanoma. This indicates that the model has a certain degree of specificity for predicting the prognosis of UVM.

## Discussion

The idea that cell death is guided by internal instructions was first suggested in 1961 in a study of insects (Tower, 2015). This landmark discovery opens the door to the study of cell death. Since then, the concept of programmed cell death has been proposed, and its significance and mechanism are being intensively explored (Nagata and Tanaka, 2017). According to existing studies, cell apoptosis, cell necrosis, and cell pyroptosis constitute the three main parts of programmed death, and relevant studies are in full bloom (Bedoui et al., 2020).

Uveal Melanoma, as one of the most harmful diseases to human vision, has seriously hindered the development of the social economy (Souto et al., 2019). Exploring the biological potential of this kind of cancer is primed and poised (Kashyap et al., 2016). Hallmarks of cancer mainly include excessive activation of growth signals, unrestricted replication, inhibition of cell death, immune re-editing, and metabolic reprogramming (Xie et al., 2021b; Xie et al., 2021c). As a kind of programmed death, cell pyroptosis plays an indelible role in both normal homeostasis regulation and cancer occurrence (Zhaolin et al., 2019). In cancer, pyroptosis has a dual effect. On the one hand, inducing pyroptosis of cancer cells can reduce tumor load in some cancers (Kesavardhana et al., 2020). Adversely, in other cancers, inflammatory substances released by cell rupture during pyroptosis can activate multiple cancer signals and may cause changes in the immune microenvironment that are beneficial for cancer proliferation (Hou et al., 2020). Hence, it’s particularly important to explore the implications of char death in particular cancer types.

In this study, we built a prognosis model related to pyroptosis in UVM. According to the risk score, UVM patients can be divided into high-risk and low-risk groups, with differences in prognosis, immune infiltration, signaling pathways, and drug sensitivity between the two groups. It can be seen that our prognostic model can accurately assess the prognosis of UVM patients. In addition, we further verified our results in cell experiments.

At present, although multi-disciplinary and multi-protocol therapies have been applied to UVM, and the research on UVM is increasing, the understanding of the pathophysiology mechanism and tumor microenvironment of UVM is far from sufficient (Li et al., 2020). Moreover, the prognosis for UVM in the current context is still poor. Early diagnosis, early treatment, and the search for new prognostic markers are urgent (Zheng and Li, 2020). We constructed the prognosis model of UVM through pyroptosis-related genes and divided UVM patients into the high-risk group and low-risk group by calculating risk scores. Such a grouping pattern can accurately predict the prognosis of UVM patients. This is beneficial for the diagnosis and treatment of UVM.

The tumor immune microenvironment is still a research focus in oncology (Junttila and de Sauvage, 2013). In this complex environment, there are interactions between multiple cells and multiple signaling molecules to promote tumor growth (Wu and Dai, 2017). The degree of infiltration of immune cells in the tumor microenvironment provides a reference for us to understand tumor immunity and tumor pathogenesis (Kim and Bae, 2016). Inflammatory substances released by pyroptosis are involved in the formation of the immune microenvironment. It can be seen that after the prognosis model of UVM was constructed by pyroptosis-related genes, there was a difference in the degree of immune infiltration between the high-risk group and the low-risk group. This not only helps us to understand the immune regulatory role of pyroptosis in UVM but also provides a reference for us to explore the differences in the immune microenvironment of UVM.

Immune checkpoint inhibitors are a major discovery in the history of cancer therapy and have been successfully used in a variety of solid tumors (Dolladille et al., 2020). Cutaneous melanoma is a typical case. Although there are differences in the pathophysiology between UVM and cutaneous melanoma, the treatment of cutaneous melanoma can provide a reference for the treatment of UVM because they both originate from melanocytes (Alavi et al., 2018). Therefore, the significance of immunotherapy in UVM is also being explored in this study. We found that the high-risk group had a higher propensity to express genes associated with immune checkpoints than the low-risk group. This may suggest that UVM patients in the high-risk group may benefit more from immune checkpoint inhibitor therapy.

In our study, ANO6 is a significant gene in the pyroptosis-related prognosis model. In addition, we verified the expression and functional effects of ANO6 in UVM through cell experiments. ANO6 has also been preliminarily described for its role in immunity. Szteyn et al. found that ANO6 is a Ca (2+) activated Cl (-) channel in mouse dendritic cells (DCs) and plays an important role in chemokine-induced DC migration (Szteyn et al., 2012). Ousingsawat et al. found that ANO6 is involved in bacterial phagocytosis and promotes THP1 macrophages to kill bacteria (Ousingsawat et al., 2015). Their study demonstrated the role of ANO6 in macrophage-associated immune defense. Seidel et al. found that ANO6 may be involved in the regulation of CD137/CD137L immune response and may have an impact on immunotherapy approaches targeting CD137 (Seidel et al., 2021). Therefore, ANO6 has potential significance in tumor immunity.

All in all, our study provides a new idea for the diagnosis, treatment, and prognosis of UVM. This has significant implications for UVM, a tumor with a poor prognosis. Future studies are expected to explore the tumor microenvironment of UVM.

## Conclusion

We constructed the prognosis model of pyroptosis related genes in UVM. Our model can accurately assess the prognosis of UVM patients. In addition, our model provides some ideas for exploring the tumor microenvironment of UVM.

## Data Availability

The raw data supporting the conclusion of this article will be made available by the authors, without undue reservation.
