# Supplementary material for: Construction and Validation of a Novel Pyroptosis-Related Gene Signature to Predict the Prognosis of Uveal Melanoma
Source: Front Cell Dev Biol. 2021 Nov 26;9:761350. doi: 10.3389/fcell.2021.761350 (PMC8662541; doi:10.3389/fcell.2021.761350)
Supplement: Supplementary file 1 [file Table1.docx]

| ANO6 | Sense5’-3’:GCAUACGAAUCUAACCUATT  Antisense5’-3’:UAAGGUUAGAUUCGUAUGCTT |
| --- | --- |
| Negative Control | Sense5’-3’:UUCUCCGAACGUGUCACGUTT  Antisense5’-3:ACGUGACACGUUCGGAGAATT |
