# Supplementary material for: Construction and Validation of a Novel Pyroptosis-Related Gene Signature to Predict the Prognosis of Uveal Melanoma
Source: Front Cell Dev Biol. 2021 Nov 26;9:761350. doi: 10.3389/fcell.2021.761350 (PMC8662541; doi:10.3389/fcell.2021.761350)
Supplement: Supplementary file 2 [file Table2.docx]

| The name of the primer | Base sequence (5 'to 3') | The number of bases |
| --- | --- | --- |
| GAPDH.F | GGAGCGAGATCCCTCCAAAAT | 21 |
| GAPDH.R | GGCTGTTGTCATACTTCTCATGG | 23 |
| ANO6.F | TGTCCCCGATTTGGGATCACT | 21 |
| ANO6.R | CGTATGCTTGTCTTTTCCTCCT | 23 |
